# Hidden but revealed: After years of genetic studies behavioural monitoring combined with genomics uncover new insight into the population dynamics of Atlantic cod in Icelandic waters

**DOI:** 10.1111/eva.13471

**Published:** 2022-09-01

**Authors:** Christophe Pampoulie, Paul Ragnar Berg, Sissel Jentoft

**Affiliations:** ^1^ Marine and Freshwater Research Institute Hafnarfjörður Iceland; ^2^ Norwegian Institute for Water Research Oslo Norway; ^3^ Department of Natural Sciences, Centre for Coastal Research (CCR) University of Agder Kristiansand Norway; ^4^ Centre for Ecological and Evolutionary Synthesis Oslo Norway

**Keywords:** behavioural ecotypes, *Gadus morhua*, genetics/genomics, Iceland, management perspective, stock structure

## Abstract

Stock structure is of paramount importance for sustainable management of exploited resources. In that context, genetic markers have been used for more than two decades to resolve spatial structure of marine exploited resources and to fully fathom stock dynamics and interactions. While genetic markers such as allozymes and RFLP dominated the debate in the early era of genetics, technology advances have provided scientists with new tools every decade to better assess stock discrimination and interactions (i.e. gene flow). Here, we provide a review of genetic studies performed to understand stock structure of Atlantic cod in Icelandic waters, from the early allozyme approaches to the genomic work currently carried out. We further highlight the importance of the generation of a chromosome‐anchored genome assembly together with whole‐genome population data, which drastically changed our perception of the possible management units to consider. After nearly 60 years of genetic investigation of Atlantic cod structure in Icelandic waters, genetic (and later genomic) data combined with behavioural monitoring using Data Storage Tags shifted the attention from geographical population structures to behavioural ecotypes. This review also demonstrates the need for future research to further disentangle the impact of these ecotypes (and gene flow among them) on the population structure of Atlantic cod in Icelandic waters. It also highlights the importance of whole‐genome data to unravel unexpected within‐species diversity related to chromosomal inversions and associated supergenes, which are important to consider for future development of sustainable management programmes of the species within the North Atlantic.

## 1 INTRODUCTION

The Atlantic cod (*Gadus morhua* L.) has been one of the most important commercial species in the North Atlantic for more than 1000 years, with evidence of cod trading during the Viking age (Star et al., 2017). Atlantic cod has been intensively exploited for the last 100 years, which led to the drastic collapse of several stocks in many regions owing to overexploitation (see Christensen et al., 2003 for a review). This was also the case in Icelandic waters where the spawning stock biomass (SSB) of Atlantic cod decreased from 1 million tonnes in the 1950s to <200,000 tonnes in the 1980s (ICES, 2019). Such a decrease is likely a result of the rapid changes in the exploitation capacities such as increase in boat size, engine power and more efficient fishing gear. Since the early 1990s, SSB increased gradually and reached 600,000 tonnes in 2018 (MFRI, 2018). Concurrently to the observed decrease in SSB from the 1950s to 1980s, significant changes were observed in the life history of Icelandic cod, indicating potential fisheries‐induced effects. Age truncation and consecutive changes in size distribution (Schopka, 1994) as well as maturity at younger ages and smaller sizes were reported (Marteinsdóttir & Begg, 2002).

**FIGURE 1:**
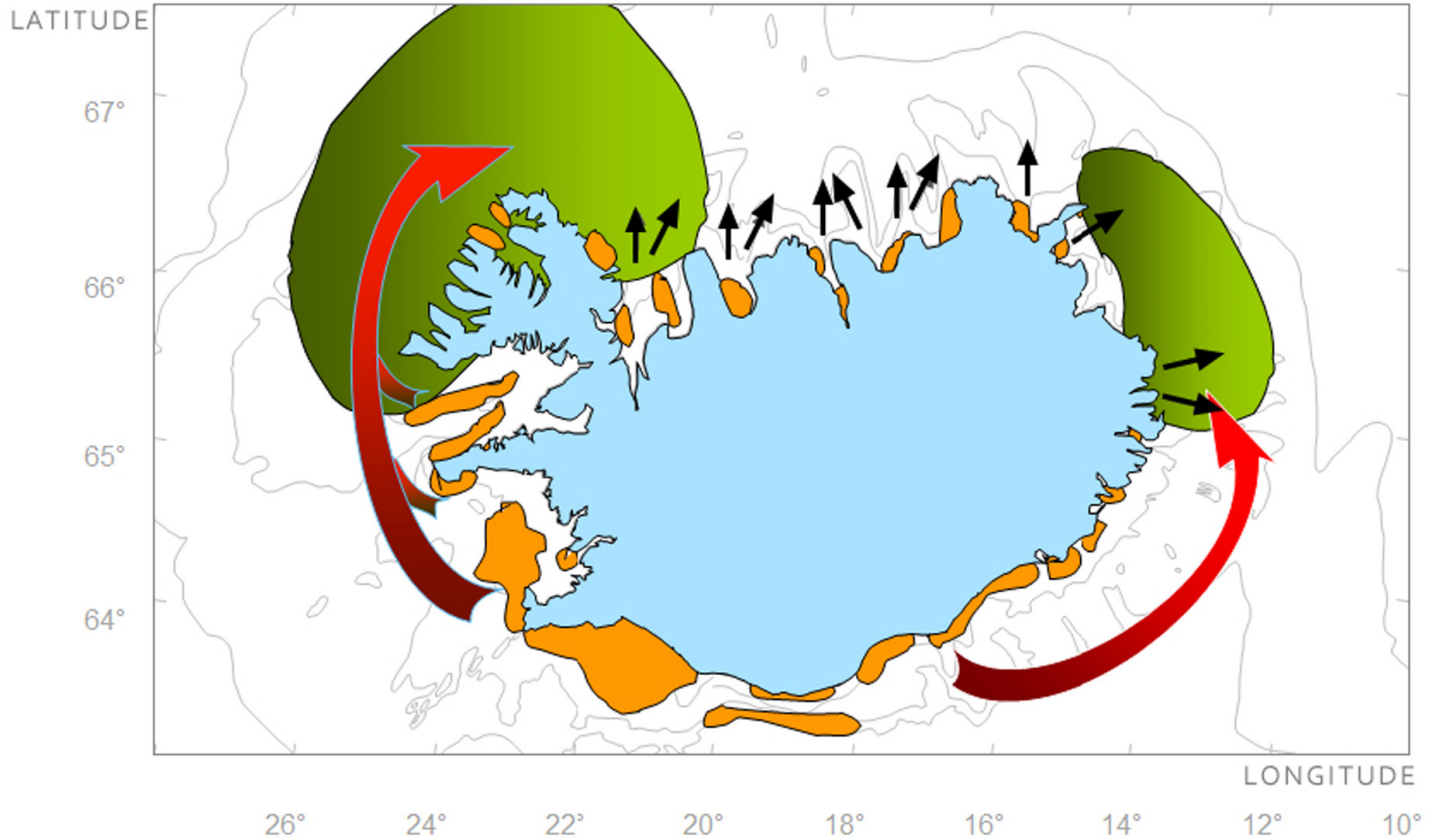
Atlantic cod migration dynamic in Icelandic waters. Spawning grounds are indicated with orange areas, while feeding ground locations are indicated in green colour. The feeding migration from the southern spawning grounds is indicated by red arrows, while black arrows indicate migration from the northern spawning grounds to more localized feeding grounds.

In Icelandic waters, the dynamic migration pattern of cod from spawning to feeding grounds was described as early as the 1700s (Magnússon, 1785) and was later confirmed by extensive tagging experiments conducted over eight decades in the 20th century. The first indication of multiple stocks of Atlantic cod in Icelandic waters was obtained from tagging experiments as early as the 1900s using Petersen tags (Sæmundsson, 1913; Schmidt, 1907), followed by successive tagging experiments in the years from 1948 to 1986 (see Jónsson, 1996). These results, spanning over a period of 200 years, were crucial for the understanding of cod dynamics in Icelandic waters and provided the first evidence for both spawning site fidelity and homing behaviour. The studies showed that postspawning cod, tagged at spawning grounds from the southwest (SW) region, undertook long‐distance migrations to one of the two main feeding regions located either (i) northwest (NW) or (ii) northeast (NE) of Iceland, respectively (Jónsson, 1996; Pálsson & Thorsteinsson, 2003; see Figure 1). Conversely, postspawning cod tagged in the northern regions were shown to be more sedentary than their SW counterpart (Jónsson, 1996) and were rarely recaptured at the main feeding grounds. The common understanding was that the majority of the Icelandic cod stock originated from one main spawning ground in the south/southwestern region (Jónsson, 1996), where the migration pattern from these spawning grounds to feeding grounds NW and NE was assumed coupled to homing behaviour. It was also known that in addition to the main spawning ground in south/southwest, spawning occurred in numerous small spawning aggregations along the coast from the NW to the southeast (SE). However, the contribution to the SSB from these smaller spawning aggregations was relatively small compared with the contribution from the main spawning ground in south/southwest (Figure 1; Jónsson, 1996; Sólmundsson et al., 2015).

Over the years, numerous studies confirmed that features of the Icelandic cod life portfolio, such as the importance of biologically relevant diversity within the species, were distinctly variable. Pronounced differences were observed between north and south of Iceland regarding age at maturity, growth rate, eggs and larval drift, otolith chemistry and many other parameters (Begg & Marteinsdottir, 2003; Brickman et al., 2007; Grabowski et al., 2011; Jónsdóttir et al., 2006a, 2006b, 2007, 2008; Marteinsdóttir & Begg, 2002; Marteinsdóttir, Guðmunðsdóttir, et al., 2000; Marteinsdóttir, Gunnarsson, et al., 2000; Pálsson & Björnsson, 2011; Pardoe et al., 2008, 2009; Pardoe & Marteinsdóttir, 2009; Pétursdóttir et al., 2006; Righton et al., 2010; Thorsteinsson et al., 2012). As such, biological evidence on the presence of several Icelandic cod populations that exhibit different life‐history traits has accumulated for nearly two decades. In the same time period, few comprehensive genetic studies were performed to assess the importance of gene flow and connectivity among the Icelandic cod populations.

Here, we review genetic studies performed on Atlantic cod in Icelandic waters, by summarizing them from the earliest haemoglobin studies performed in the 1960s to recent genomic investigations. We focus specifically on studies investigating genetic population structure and we describe how the advancement of genomic tools has changed our perception of biological units in the last six decades. Based on new evidence from whole‐genome sequencing, combined with Data Storage Tags (DSTs), the behavioural ecotypes (coastal and frontal ecotypes) might play a crucial role in the maintenance of the described spatial genetic structure in Icelandic waters. These potential new management units revealed in Icelandic waters represent parallel evolutionary adaptive cod lineages that were recently found across the species range (Matschiner et al., 2022) and represent crucial information that should be taken into consideration for the development of sustainable management programmes for Atlantic cod stocks through its distribution range.

## 2 THE GENETIC PIONEERS: STUDIES OF HAEMOGLOBIN POLYMORPHISM

Genetic studies of stock structure in Icelandic waters started as early as the 1960s, when Sick (1965) revealed the presence of limited genetic variation at the haemoglobin *HbI* locus. Several years later, Jamieson and Birley (1989) were the first ones to describe a clear difference between cod from the NE and the SW of Iceland, owing to a frequency shift of the *HbI* ^*1*^ allele from 0.61 in the NE to 0.09–0.32 in the SW. Although very little was known about the haemoglobin variants in the early 1980s, these findings have now been confirmed and two amino‐acids replacements, Met55‐β_1_Val and Lys62‐β_1_Ala located at crucial positions in the α_1_β_1_ subunit interface and haem pocket, respectively, were discovered (Andersen et al., 2009). The described *HbI* variants seemed to affect the oxygen‐binding properties differently in various cod populations and were assumed to reflect adaptation to local environmental conditions (Andersen et al., 2009). Further, evidence of the multi‐copy nature and potential adaptive significance of haemoglobin has accumulated and questioned the functionality of the different haemoglobin variants (Baalsrud et al., 2017; Barlow et al., 2017; Borza et al., 2009; Knight, 2017). By doing these early investigations, Jamieson and Birley (1989) nevertheless presented the first evidence of local adaptation in a species with large population sizes and potentially high gene flow. As such, they were the pioneers in a long series of studies investigating the potential population structure of Atlantic cod in this region. However, these results remained unnoticed for many years and were not supported by the first sequence variation studies of mitochondrial DNA (mtDNA; Árnason & Rand, 1992; Árnason et al., 1992, 2000). By investigating mtDNA sequence variation in Iceland and Greenland, these studies reported a high degree of variation and suggested a lack of differentiation of haplotype frequencies within the studied regions, that is, a lack of genetic structure. Subsequently, the Icelandic cod stock was thought to be composed of a single management unit for many years (Schopka, 1994).

## 3 THE PANTOPHYSIN (*PAN* I LOCUS) ERA

One of the most popular genetic markers for Atlantic cod in the Northeast Atlantic was the synaptophysin locus described by Fevolden and Pogson (1997), later called the pantophysin locus (*Pan* I). The popularity of the *Pan* I locus lasted more than a decade and was probably linked to the fact that this gene contains two major alleles, *Pan* I^A^ and *Pan* I^B^, and to the ease of obtaining genotypes. The two alleles *Pan* I^A^ and *Pan* I^B^ differ by six fixed nonsynonymous DNA substitutions, clustering in the first intravesicular loop (IV1 domain) of the protein (Pogson & Mesa, 2004). The function of this locus is still poorly understood, but it has been suggested to be a potential candidate gene under selection (Pogson & Mesa, 2004). The gene codes for an integral membrane protein expressed in cytoplasmic transport vesicles (Brooks et al., 2000; Windoffer et al., 1999).

In the following years, multiple studies revealed differences in *Pan* I allele frequencies across the North Atlantic Ocean and potential driving forces behind the selection at this locus were investigated (Case et al., 2005; Karlsson & Mork, 2003; Pampoulie et al., 2006; Pogson, 2001; Sarvas & Fevolden, 2005a; Skarstein et al., 2007; Stenvik et al., 2006). In Icelandic cod, differences in *Pan* I allele frequencies were observed at relatively small geographical scales in the SW region and were shown to be temporally stable over a period of 2 years (Imsland et al., 2004; Jónsdóttir et al., 1999, 2001). However, 10 more years passed before information on *Pan* I allele frequency variation across the entire spawning regions of Atlantic cod in Icelandic waters were available (Pampoulie et al., 2006). By collecting and genotyping more than 2500 spawning cod at 22 different locations around Iceland, using the *Pan* I locus and nine microsatellite loci, Pampoulie et al. (2006) were the first to demonstrate that Icelandic cod populations were not panmictic, but consisted of at least two genetically differentiated spawning components, the NE and SW. They observed a distinct *Pan* I allele shift between these two regions with a higher frequency of the *Pan* I^B^ allele in the southwestern spawning ground compared with the northern region. The observed difference in *Pan* I^B^ allele frequencies was also supported by differentiation at microsatellite loci and by tagging experiments. Nevertheless, the level of differentiation observed with the *Pan* I locus was 80‐fold higher than the one observed for microsatellite loci, a result interpreted as evidence for potential local adaptation. Moreover, Pampoulie et al. (2006) confirmed the increase in *Pan* I^B^ allele frequency linked to the depth in which cod occur and which had been previously observed in the SW region (Jónsdóttir et al., 1999). The concept of an inshore versus offshore cod population within the region emerged.

While these results were questioned by a subsequent study (Eiríksson & Árnason, 2013), the debate was mainly centred around the acceptance of the conclusion drawn about a NE–SW structure in the Icelandic cod and about the consideration of selective processes (adaptation) in conservation and management practices. Today it is generally accepted that natural selection (local adaptation) processes are important to identify population structure and should be used within a management context to conserve within‐species diversity (Nielsen et al., 2009). Although the mechanisms behind the observed allele frequencies at the *Pan* I locus are still not completely understood, a majority of the subsequent genetic/genomic studies confirm the presence of unique NE versus SW units as well as a distinction linked to the depth cod occurs as proposed by Pampoulie et al. (2006). The *Pan* I locus remains one of the most used genetic markers to study population structure in Atlantic cod, and it is now clear that the locus is located within a large inverted genomic region at linkage group 1 (LG1), known to discriminate between the iconic migratory Northeast Arctic cod (NEAC) and the stationary Norwegian coastal cod (NCC; Berg et al., 2016, 2017). This chromosomal inversion contains hundreds of genes, each of which might play an important role in driving local adaptation, and therefore renders the discussion about the adaptive role of the *Pan* I locus speculative. It is highly likely that it is this collection of linked genes, defined as a supergene inherited in a Mendelian manner, rather than a single gene which is driving the adaptive process of cod to local environmental conditions.

## 4 SINGLE‐NUCLEOTIDE POLYMORPHISMS (SNP MARKERS)

In the early 2000s, the advent of new genetic technology introduced novel genetic markers that enabled further investigations of genetic structure in Icelandic cod. As such, the development of next‐generation sequencing and hence of thousands of SNPs made a significant impact in many nonmodel organisms such as cod (Bonanomi et al., 2015, 2016; O'Leary et al., 2006; Therkildsen et al., 2013; Wirgin et al., 2007). By utilizing these novel techniques, several studies suggested that ecological divergence (in the presence of gene flow) was pronounced and affected specific genomic regions, so‐called ‘genomic islands of divergence’ (Bradbury et al., 2013; Hemmer‐Hansen et al., 2013) (previously referred as heterogeneous genomic divergence; see Nosil et al., 2009), whereas the remaining parts of the genome were homogenized by gene flow. Several SNP‐based studies that were not focusing exclusively on the genetic structure of cod in Icelandic waters confirmed the discrimination between the NE and SW breeding grounds (Bonanomi et al., 2015; Therkildsen et al., 2013). Altogether, the novel SNP‐based studies confirmed the presence of two distinct Atlantic cod populations in Icelandic waters where differentiation was mainly driven by selective processes at key genomic regions. Using 1152 validated transcriptome‐derived SNPs, Therkildsen et al. (2013) confirmed the previously described inshore versus offshore cod population differences in the SW region of Iceland (Jónsdóttir et al., 1999; Pampoulie et al., 2006).

## 5 DATA STORAGE TAGS PROVIDE CRITICAL NEW INFORMATION

While geneticists were focussing on potential spatial genetic structure of Atlantic cod in Icelandic waters, marine biologists were trying to better understand the role of this species in the food web and its potential migration routes. Two pioneers drastically changed the perception of stock structure and questions related to conservation and management of the Icelandic cod and consequently also the path of genetic investigation in Icelandic waters for the following 20 years. In the early 2000s, Pálsson and Thorsteinsson (2003) conducted DSTs experiments on a spawning ground at the southwestern coast of Iceland from 1996 to 1999. DSTs are biologging devices, which are introduced in the abdominal cavity of individual fish where they record depth and temperature with high accuracy at a constant time interval.

**FIGURE 2:**
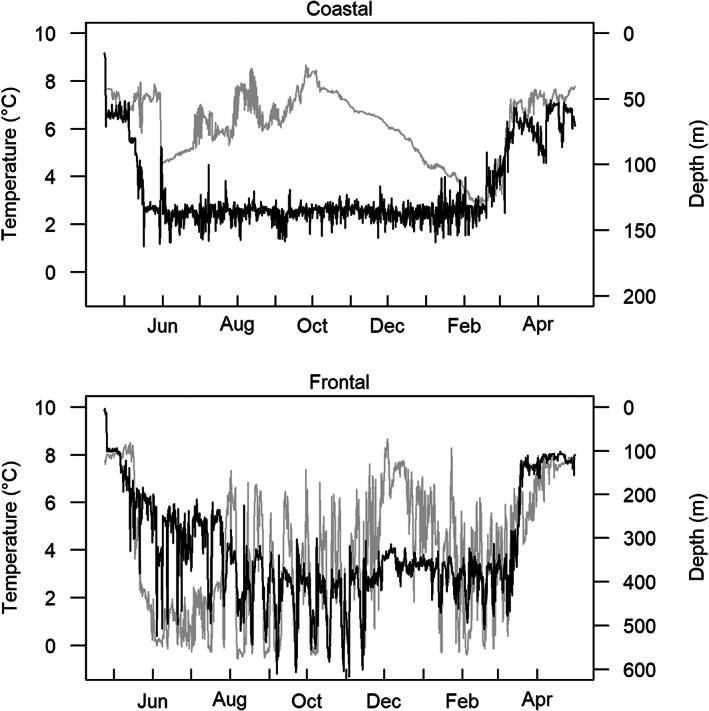
Typical coastal (upper) and frontal (lower) data storage tags profiles. Depth is depicted in black, temperature in light grey.

The results of the DSTs experiment were quite surprising. Among individuals tagged within the same inshore spawning location, some individuals appeared to stay all year in shallow coastal waters (<200 m), characterized by seasonal trend in temperature (abbreviated ‘coastal cod’), while other made feeding migrations to deeper waters where they foraged in thermal fronts (abbreviated ‘frontal cod’; Figure 2).

Additionally, the use of DSTs provided evidence of spawning skippers (e.g. mature cod which do not reproduce during one spawning season, see Jónsdóttir et al., 2014), as well as evidence of the fact that: (a) the migration timing in successive years was close to being synchronous, suggesting that the onset of migration was consistent, (b) the use of a tidal model suggested that different behavioural types were undertaking feeding migration in groups or shoals, and (c) the stability of behaviour from year to year suggested that the behavioural strategies were related to food availability or genetic differences (Thorsteinsson et al., 2012).

Similar distinguishable migration patterns were described as early as the 1930s in Norwegian waters, where two distinct Atlantic cod ecotypes were described, the NEAC and the NCC (Rollefsen, 1934). NEAC is known to exhibit long‐distance migrations from feeding areas located in the Barents Sea and the Svalbard region (Bergstad et al., 1987) to its spawning grounds along the Norwegian coast where the main spawning areas are located around the Lofoten Islands (Bergstad et al., 1987; Brander, 1994). Such a migratory pattern is similar to what is observed for the frontal Icelandic cod. The NCC are more stationary and usually resident in more shallow and warmer waters along the Norwegian coastline including the Lofoten Islands (Rollefsen, 1954) and in numerous fjords in which they usually spawn (Jakobsen, 1987). Hence, the NCC display a migration pattern that is similar to what is observed in the coastal Icelandic cod.

## 6 BEHAVIOURAL ECOTYPES EXHIBIT GENETIC DIFFERENCES AT THE PANTOPHYSIN AND RHODOPSIN GENES

The first attempt to understand the genetic background of coastal and frontal behavioural ecotypes in Icelandic waters utilized the most common genetic marker for Atlantic cod at the time, the *Pan* I locus. Based on data collected from 69 DSTs‐recaptured individuals, Pampoulie et al. (2008) showed that 97% of *Pan* I^AA^ genotypes exhibited a typical coastal behaviour, while 88% of *Pan* I^BB^ genotypes exhibited a frontal behaviour. The heterozygotes *Pan* I^AB^ exhibited either coastal or frontal behaviours with a 50%–50% proportion, which implied that using the *Pan* I locus alone was not sufficient to accurately assign individual cod to behaviour ecotypes in this region.

**FIGURE 3:**
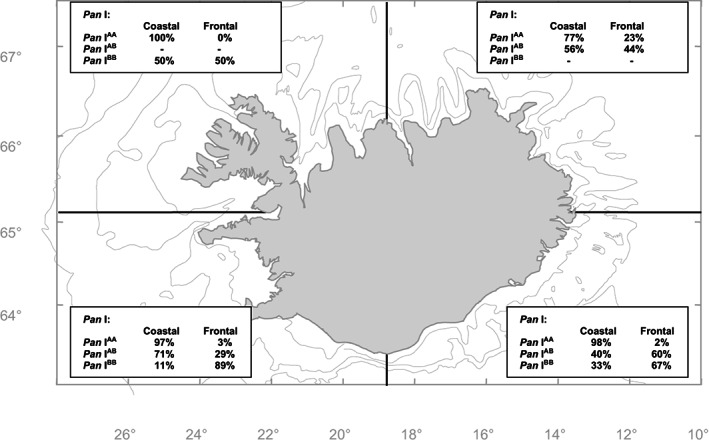
Proportion of the different *Pan* I genotypes among the coastal and frontal ecotypes within geographical regions during spawning time (data analysed for this review, *n* = 172).

Further analyses considering geographical partitioning of behavioural ecotypes, using a higher number of recaptured individuals (*n* = 172), demonstrated that almost no *Pan* I^BB^ genotypes were captured in the north and that the relationship between the *Pan* I genotypes and the behavioural ecotypes varied among regions (Figure 3). While most of the *Pan* I^AA^ genotypes were of the coastal ecotype in the west and southeast of Iceland, 23% of the recaptured ones of the northeast exhibited a frontal behaviour (Figure 3). The same pattern was observed with the *Pan* I^BB^ genotypes for which only 67% of them exhibited a frontal behaviour in the southeast compared to 89% in the southwest. The *Pan* I^AB^ genotypes also exhibited a higher percentage of frontal behaviour in the southeast than in any other region. Interestingly, similar associations between behavioural ecotypes and the *Pan* I locus allele frequencies were also demonstrated among NEAC and NCC in Norwegian waters (Nordeide, 1998; Sarvas & Fevolden, 2005a, 2005b; Skarstein et al., 2007).

Since these ecotypes are occurring at different depths during most of the year, they are clearly exposed to different light conditions. In the rhodopsin pigment, which is involved in light detection, several amino acid substitutions have been shown to affect the spectral sensitivity in several teleost species (Yokoyama et al., 2008). Numerous studies provide evidence of the importance of protein modifications of rhodopsin in marine vertebrates, resulting in local adaptation to various light environments and ultimately to species diversification (Ebert & Andrew, 2009; Shum et al., 2014; Sivasundar & Palumbi, 2010). Consequently, Pampoulie et al. (2015) focussed on the polymorphism in the *RH1* opsin gene, using 148 tagged and recaptured individuals with DSTs, and observed 18 variable sites within the *RH*1 opsin gene, and two in the 3′‐untranslated region (3′‐UTR). However, only two of these polymorphic sites had high MAFs that markedly differed between behavioural ecotypes (one synonymous SNP at site 459 [AA153] and one nonsynonymous at site 1295 in the 3′‐UTR).

For nonmodel organisms such as Atlantic cod, the genomic era offered ample opportunity to better understand genomic features such as the observed *Pan* I locus and the *RH*1 opsin gene variation among the behavioural ecotypes. Both these genes were shown to be located within the large chromosomal inversion on LG1, and as mentioned above found to be involved in behavioural ecotypes divergence in the North Atlantic (Berg et al., 2017).

## 7 ENTERING THE GENOMIC ERA AND AN EVALUATION OF POTENTIAL MANAGEMENT UNITS

**FIGURE 4:**
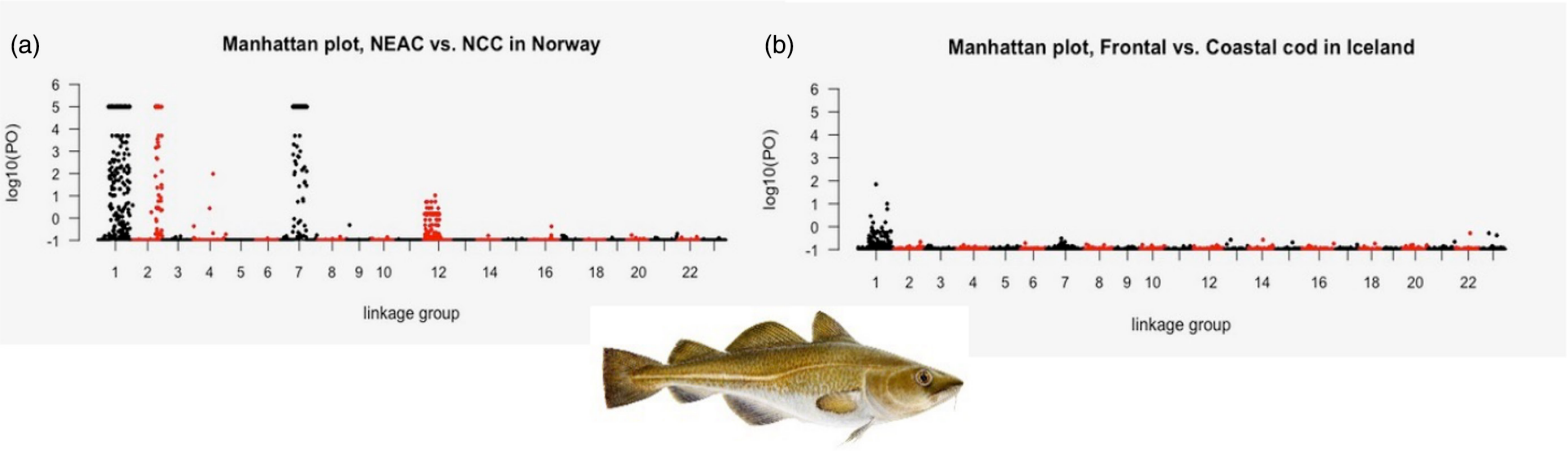
Majority of detected outliers' loci within the Atlantic cod genome are clustered within linkage groups (LGs) 1, 2, 7 and 12 for the migratory and nonmigratory cod including the NEAC/NCC complex (a) and the Icelandic coastal and frontal behavioural ecotypes (b) described using DSTs data (reanalysis of data from Berg et al., 2016, 2017). Cod drawing was provided by Jón Baldur Hlíðberg©. DSTs, Data Storage Tags; NCC, Norwegian coastal cod; NEAC, Northeast Arctic cod.

The Atlantic cod was one of the first nonmodel organism for which a chromosome‐anchored draft genome assembly was available (Star et al., 2011). However, it took another 5–6 years before the first whole‐genome study in Icelandic waters was performed. The study was performed on samples collected on a large geographical scale to understand genome‐wide patterns of divergence among the behavioural ecotypes of Atlantic cod (Berg et al., 2017). The population‐based sequencing efforts in Atlantic cod identified genome‐wide patterns of divergence—mainly linked to four large chromosomal inversions—shedding light on processes of local adaptation in spatially structured populations across the North Atlantic Ocean (Berg et al., 2017). It was shown that three of these genomic regions—on LG1, LG2 and LG7—clearly discriminated the migratory NEAC from the nonmigratory NCC as well as the coastal and frontal ecotypes found around the Icelandic waters and characterized by DSTs profiles (Berg et al., 2016, 2017; Figure 4). The chromosomal inversions, or supergenes, which span several Mb and contain hundreds of genes, are likely maintained by selection processes, and due to low recombination between the inversion variants, impacting the entire genomic region(s). Hence, they facilitate coevolution of genes underlying complex traits of behavioural ecotypes (Berg et al., 2017) such as the *Pan* I locus and the *RH1* gene both located within the large chromosomal inversion in LG1 (as mentioned above).

Moreover, the genomic data also indicated that the migratory ecotype NEAC originated from the stationary ancestral ecotype NCC. The derived inversion variant found on LG1, the main inversion linked to the behavioural difference between the NEAC and NCC (Berg et al., 2016; Hemmer‐Hansen et al., 2013; Kirubakaran et al., 2016), is found in high frequency (0.50) in NEAC, whereas the ancestral inversion variant is most frequently observed (almost fixed: 0.93) in the NCC and other cod populations (Berg et al., 2017; Matschiner et al., 2022). However, a clear separation (i.e. genetic differentiation) between NEAC and NCC is also found within the inversions on LG2 and LG7 (Berg et al., 2017). These inversions seem to also vary more in frequency linked to environmental conditions (Barth et al., 2019; Berg et al., 2015; Kirubakaran et al., 2020) Thus, the separation seen here on LG2 and LG7 between NEAC and NCC could be due to the fact that NEAC is experiencing more extreme and colder environmental conditions (Berg et al., 2015). For the Icelandic coastal and frontal behavioural ecotypes, however, the allele frequency differences showed a higher degree of complexity. Even if there is seemingly a differentiation between the coastal and frontal behavioural ecotypes in terms of the inversion found on LG1 (see Figure 4), the genetic differentiation is less pronounced than for the Norwegian counterparts (Berg et al., 2017). This is mainly due to a less clear separation in the inversion frequencies found between the two ecotypes, with most of the frontal cod being either heterozygous (0.69) or homozygous (0.21) for the derived inversion variant, whereas the coastal cod displayed a higher frequency of the ancestral variant (0.59), and some heterozygote individuals (0.33) were also detected (Berg et al., 2017). For the two other inversions (on LG2 and LG7), the separation between Icelandic coastal and frontal behavioural ecotypes was not that obvious at all (see Figure 4 and supp. material of Berg et al., 2017). Both the frontal and coastal behavioural ecotypes had higher frequencies of the inversion variant(s) dominating in the migratory NEAC; 0.76 and 0.52, respectively, for the inversion variant on LG2 and 0.77 and 0.55, respectively, for the inversion variant on LG7 (see supp. material of Berg et al., 2017). These results indicate quite strongly that the two behavioural ecotypes found in Icelandic waters have most likely derived from NEAC (Berg et al., 2017). This is supported by less genetic differentiation observed between coastal and frontal behavioural ecotypes compared with the differentiation detected in the two ecotypes in the Norwegian waters as supported by the majority of outliers loci (Figure 4). These observations could be linked to higher complexity of behaviour differentiation in the Icelandic waters.

Genomic diversity within species is as important as diversity of species for ecosystem function (Hoban et al., 2022), and the presence of genomic regions of divergence among behavioural ecotypes of Atlantic cod highlights the importance of full genome data for biodiversity conservation and management. Thus, further research is warranted, to fully pinpoint the genomic signatures underlying behavioural ecotypes, as well as how these behavioural differences and migration patterns (via gene flow as well as genetic drift) impact the population structure of Atlantic cod in Icelandic waters. Such information is of high value for future development of sustainable management programmes of these important fish stocks. As mentioned above, in other geographical regions differentiation in supergene frequencies has been shown to be correlated with various environmental characteristics such as seawater temperature (Barney et al., 2017) and salinity (Berg et al., 2015) and to promote ecological stasis and persistence over millennia despite the fisheries‐induced decline in populations (Sodeland et al., 2022).

## 8 CONCLUSION AND PERSPECTIVES

One of the premises of scientific results integration into management plans and conservation practices is to fully fathom diversity within the distribution range of a harvested species. Several distinct approaches have been used for decades, and the last consensus has been that multidisciplinary approaches should be developed. Molecular ecology (in the large sense) should ideally join behavioural ecology, life‐portfolio diversity and other disciplines to ensure an improved knowledge of diversity within the harvested species and develop efficient plans for conservation. The DSTs experiment of Pálsson and Thorsteinsson (2003) and the consecutive single‐gene and genomic studies revealed a concealed within‐species diversity in the Icelandic Atlantic cod, which is potentially important for the ecosystem function. Hence, 60 years of investigation of genetic structure of cod in Icelandic waters drastically changed our perception of potential management units. The interest moved from geographical differences mentioned as early as the 1980s to an increasing interest in hidden within‐species diversity in the form of behavioural ecotypes, which is reflected in several genomic studies and revealing the presence of four large chromosomal inversions potentially responsible for local adaptation.

Nevertheless, the story of the Icelandic cod stock genetic structure is far from being fully understood. At present, and before any management measures are taken, there is an urgent need to assess the relative role of the behavioural ecotypes versus the observed geographical partitioning (Southwest vs. Northeast) in maintaining the observed stock structure in Icelandic waters. While genomics has contributed to a better knowledge of the within‐species diversity in Icelandic cod and on the evolutionary processes behind it (Matschiner et al., 2022), further investigation is needed on the distribution of the behavioural ecotypes and their connectivity at the different spawning grounds in Icelandic waters.

At present, the Icelandic cod is still managed as a single stock despite the burgeoning literature pointing out to the large variation in genetic and life‐history traits. Fisheries are partially closed during spawning time (e.g. on spawning grounds), but no real measures are taken on the feeding grounds. In addition, despite the numerous structure studies performed in Iceland, several crucial questions remain, and it is therefore premature to draw conclusion on the dynamic of the cod stock in this region. The primary remaining question to resolve is to understand the role of the inverted variants in the maintenance of geographical versus ecotypes divergence both during spawning time (population differentiation) and during the rest of the cod life cycle (contribution to nursery, juveniles and feeding aggregations). Once this crucial question is resolved, the temporal stability of the observed structure and the effect of fisheries can be investigated further.

To conclude, the advancement of genome sequencing technologies in the last decades has drastically redirected genomic investigations in nonmodel organisms, such as the Icelandic cod behavioural ecotypes. The recent use of reference genomes of coastal versus frontal Icelandic cod and of stationary versus migratory individuals of cod across the North Atlantic has confirmed the presence of supergenes under natural selection, which shaped the architecture of local adaptation of the species in Icelandic waters for the last 30,000 years and in the North Atlantic for the last 0.4–1.66 million years (Matschiner et al., 2022). Finally, this review also demonstrates the importance of reference genomes to detect the presence of an unexpected within‐species diversity related to large inverted variant(s). Large chromosomal inversions have now been successfully identified in a multitude of species and have all been shown to be related to within‐species diversity reflecting local adaptive processes (Akopyan et al., 2022; Ayala et al., 2013; Berg et al., 2015; Huang et al., 2020; Koch et al., 2021; Sanchez‐Donoso et al., 2022; Twyford & Friedman, 2015) and are therefore becoming relevant for management and for conservation genomics (Formenti et al., 2022).

## CONFLICT OF INTEREST

The authors declare no competing interests.

## Data Availability

Data reviewed in the present article were published in previous manuscripts. If not open‐access, the data can be requested to the first author of this article.
